# LncRNA PVT1 Facilitates Phenotypic Switching of Vascular Smooth Muscle Cells in Intracranial Aneurysms by Recruiting KDM1A to Reduce H3K9me2 Modification

**DOI:** 10.1002/kjm2.70025

**Published:** 2025-04-28

**Authors:** Yu‐Fei Cheng, Wei Sun, Yi‐Xia Wang, Jia‐Jie Chen, Ya‐Sen Cao

**Affiliations:** ^1^ Yangzhou University Medical College, Jiangsu Key Laboratory of Experimental & Translational Non‐Coding RNA Research, Institute of Translational Medicine Yangzhou University Yangzhou China

**Keywords:** ALOX5, intracranial aneurysms, KDM1A, LncRNA PVT1, vascular smooth muscle cells

## Abstract

This study explores the differential expression of lncRNA PVT1 in intracranial aneurysms (IAs) and its role in the phenotypic switching of vascular smooth muscle cells (VSMCs). PVT1 and ALOX5 expressions in the serum of IA patients and healthy controls were detected by qRT‐PCR. The diagnostic efficacy of PVT1 for IA was analyzed using the receiver operating curve (ROC). VSMCs were treated with H_2_O_2_ to induce phenotypic switching. PVT1 expression was silenced in VSMCs, followed by the detection of cell viability, inflammatory factors (TNF‐α, IL‐1β, IL‐6), as well as α‐SMA, OPN, and MMP2. RIP confirmed the binding between PVT1 and KDM1A. The enrichment of KDM1A and H3K9me2 on ALOX5 promoter was analyzed by ChIP. PVT1 expression in IA patients was significantly elevated, and PVT1 expression was correlated with the size and location of IAs. H_2_O_2_ induced VSMCs to transition from contractile cells to synthetic cells, intensified cellular inflammation, and upregulated PVT1 expression. Inhibition of PVT1 repressed VSMC phenotypic switching. PVT1 promoted ALOX5 expression by recruiting KDM1A to reduce H3K9me2 modification. In conclusion, PVT1 is highly expressed in IA patients and has high diagnostic value. PVT1 promotes VSMC phenotypic switching by recruiting KDM1A to reduce H3K9me2 modification and enhance ALOX5 expression.

## 1 Introduction

Intracranial aneurysm (IA) is a pathological dilation of blood vessels in a brain region, which occurs when the intracranial blood vessel walls become fragile and form bulges. The rupture of IAs can cause subarachnoid hemorrhage and portend significant mortality [1]. Currently, surgical clipping and endovascular coiling are the primary treatments for IAs, while these invasive treatment modalities may also eventuate severe complications [2]. The pathophysiology of IA formation relates to intricate mechanisms including genetic susceptibility, hemodynamic stress, chronic inflammation, and vascular remodeling [3]. Vascular smooth muscle cells (VSMCs) assume critical roles in maintaining normal vascular morphology and blood pressure. VSMCs have high plasticity and exhibit significant phenotypic switching in response to stimuli [4, 5]. The pro‐inflammatory synthetic phenotype of VSMCs may lead to extracellular remodeling, eventually resulting in the formation and rupture of IAs [4, 6]. Understanding the impact of phenotypic switching of VSMCs underlying the nosogenesis of IAs can provide new prevention and therapeutic strategies for IAs.

Long non‐coding RNAs (LncRNAs) are universally defined as 200 nucleotide extended transcripts with limited protein‐coding ability, which function as vital regulators in multiple cellular processes [7]. LncRNAs modulate phenotypic switching of VSMCs, inflammation activation, extracellular matrix (ECM) destruction, endothelial dysfunction, and excessive reactive oxygen species production, thereby affecting the formation, growth, and rupture of IAs [8, 9]. One of such lncRNAs, plasmacytoma variant translocation 1 (PVT1) can impact the development of IAs by interacting with the HOTAIR‐miR‐143‐COL1A1/COL5A2 axis [10]. PVT1 has also been identified as a differentially expressed lncRNA associated with ferroptosis in IA formation [11]. Knockout of PVT1 can reverse angiotensin II‐induced changes in abdominal aortic aneurysm in mice, including the suppression of VSMC apoptosis and ECM destruction [12]. However, the exact mechanism of PVT1 mediating phenotypic switching of VSMCs in IAs remains unknown.

Histone methylation as the most prevalent epigenetic modification is crucial for transcriptional activation and inhibition, heterochromatin‐mediated transcriptional silencing, DNA repair, and X chromosome inactivation [13]. Additionally, aberrant alterations in histone methylation markers indicate the development of brain aneurysm and subarachnoid hemorrhage [14]. The lysine‐specific histone demethylase 1A (KDM1A) is the first identified histone lysine demethylase that catalyzes the demethylation of histone H3, H3K4me1/2, and H3K9me1/2 in a context‐dependent manner [15]. KDM1A is upregulated in IAs and induces IA formation and rupture by mediating phenotypic switching of VSMCs [16]. KDM1A inhibition prevents angiotensin II‐stimulated VSMCs switching from a contractile to a synthetic phenotype [17]. Moreover, the reduction of dimethylation of histone H3 on lysine 9 (H3K9me2) enhances the binding of NF‐κB and AP‐1 at specific inflammatory response genes, thereby augmenting pro‐inflammatory stimuli in VSMCs [18]. Based on the above findings, we speculate that PVT1 facilitates VSMC phenotypic switching by recruiting KDM1A to reduce H3K9me2 modification. This study aims to investigate the differential expression of PVT1 in IAs and the mechanism of PVT1 modulating VSMC phenotypic switching, thus providing novel targets for the diagnosis and treatment of IAs.

## 2 Materials and Methods

### 2.1 Ethics Statement

The informed consent was obtained from all participants, and the study procedure was approved by the Ethics Committee of our hospital.

### 2.2 Study Subject and Sample Collection

This study prospectively recruited UIA (unruptured IA) patients who underwent microsurgical clipping at our hospital from December 2021 to December 2023. The individuals who underwent physical examinations at the health management center were selected as health controls (HCs). The inclusion criteria for the HC group are: (1) matching the age, gender, and other medical history of recruited patients; (2) no inflammation‐related diseases, cerebrovascular‐related diseases, history of various tumors, and immunological diseases.

The inclusion criteria for patients with IAs: (1) sudden headache, eyelid droop, dizziness, or diplopia, diagnosed as IAs by digital subtraction angiography; exclusion criteria: (1) patients with multiple UIAs; (2) interstitial, fusiform, traumatic, and infectious UIAs; (3) received treatment before admission; (4) combined with other cerebrovascular diseases (such as cerebral vascular malformation, smoke disease, etc.).

The elbow vein blood (3 mL) was collected from all subjects. The blood samples were left to stand for 1 h and centrifuged at 2000 g for 10 min. The serum was collected in RNase‐free centrifuge tubes, sealed and numbered, and immediately stored at −80°C.

### 2.3 Quantitative Real‐Time Polymerase Chain Reaction

The total RNA was extracted using TRIzol reagent (15596026CN, Invitrogen, Carlsbad, CA, USA) and synthesized to cDNA using RevertAid RT reverse transcription kit (K1691, Thermo Fisher Scientific, Waltham, MA, USA). Real‐time PCR was performed using AceQ qPCR SYBR Green Master Mix kit (Q111‐02, Vazyme, Nanjing, China) on the 7300 Real‐Time PCR system (Applied Biosystems, Carlsbad, CA, USA). The relative expression of genes was quantified using the 2^−ΔΔCt^ method [19], with glyceraldehyde‐3‐phosphate dehydrogenase (GAPDH) as the internal reference. Primer sequences are shown in Table 1.

**TABLE 1:** qPCR primers.

	Forward primer (5′‐3′)	Reverse primer (5′‐3′)
PVT1	TGGAATTCCACTTACGGGCC	TTCCACCAGCGTTATTCCCC
KDM1A	CACCAGCCGTTCAGTTTGTG	GCCAACATGCCCGAACAAAT
ALOX5	TGCCAACAAAACAGACCCCT	TGGTTGAGCTGGATGGCAAT
ALOX5 promoter	GACAACCGGACATGCACATG	CGCTTGTGATTTTGGGGTCC
α‐SMA	CAGCTACGTGGGTGACGAAG	ATGCTCTTCAGGGGCAACAC
OPN	CAGCTTTACAACAAATACCCAGATG	GACTTACTTGGAAGGGTCTGTG
MMP2	AGCATGTCCCTACCGAGTCT	AAACAGATGGCAAACACGGC
U6	AAAATTTCTCACGCCGGTATTC	CCTGCAGACCGTTCGTCAA
GAPDH	ACAGCCTCAAGATCATCAGC	GGTCATGAGTCCTTCCACGAT

Abbreviations: α‐SMA: α‐smooth muscle actin; ALOX5: arachidonate lipoxygenase 5; GAPDH: glyceraldehyde‐3‐phosphate dehydrogenase; KDM1A: lysine‐specific histone demethylase 1A; MMP2: matrix metalloproteinase 2; OPN: osteopontin; PVT1: plasmacytoma variant translocation 1.

### 2.4 Cell Culture and Treatment

Human cerebral artery VSMCs were purchased from Procell (CP‐H116, Wuhan, China) and cultured in Dulbecco's modified Eagle's medium (15140148, Gibco, Grand Island, NY, USA) containing 10% fetal bovine serum (A5670701, Gibco) and 1% penicillin–streptomycin (12491015, Gibco) at 37°C with 5%CO_2_. The cells were treated with 0.5 mM H_2_O_2_ [20] for 24 h to establish a cell model.

### 2.5 Cell Transfection

siRNA targeting PVT1 (si‐PVT1‐1, si‐PVT1‐2), siRNA targeting KDM1A (si‐KDM1A‐1, si‐KDM1A‐2), and si‐NC, as well as ALOX5 overexpression plasmid (oe‐ALOX5) and oe‐NC were provided by GenePharma (Shanghai, China). The above RNA and plasmid were transfected into cells using Lipofectamine 2000 reagent (11668500, Invitrogen). After 48 h of transfection, the cells were collected to detect transfection efficiency.

### 2.6 Cell Counting Kit‐8 Assay

The cells were seeded into the 96‐well plate (5 × 10^3^ cells). After 48 h, each well was added 10 μL cell counting kit‐8 (CCK‐8) reagent (CK04, Dojindo Laboratories, Japan) for 3 h of incubation. The absorbance at 450 nm was measured by a microplate reader (BioRad, Hercules, CA, USA).

### 2.7 Enzyme‐Linked Immunosorbent Assay

The contents of tumor necrosis factor‐α (TNF‐α), interleukin (IL)‐1β, and IL‐6 in cells were tested using the TNF‐α enzyme‐linked immunosorbent assay (ELISA) kit (ab181421, Abcam, Cambridge, MA, USA), IL‐1β ELISA kit (ab214025, Abcam), and IL‐6 ELISA kit (ab178013, Abcam).

### 2.8 Western Blot

VSMCs were lysed using radio‐immunoprecipitation assay buffer (P0013B, Beyotime, Shanghai, China), and the protein concentration was detected using a bicinchoninic acid assay kit (P0012, Beyotime). An equal amount of protein was purified by SDS‐PAGE and then transferred onto polyvinylidene fluoride membranes. The membranes were blocked with skim milk at 25°C for 1 h and incubated with the primary antibodies KDM1A (1:10,000, ab129195, Abcam), ALOX5 (1:1000, ab169755, Abcam), α‐smooth muscle actin (α‐SMA) (1:1000, ab5694, Abcam), osteopontin (OPN) (1:1000, ab214050, Abcam), matrix metalloproteinase (MMP2) (1:2000, ab92536, Abcam), and α‐tubulin (1:5000, ab52866, Abcam) at 4°C overnight. Afterward, the membranes were washed with PBS twice and incubated with the secondary antibody IgG (1:5000, ab205718, Abcam) at 25°C for 1 h. Finally, the specific protein bands were visualized using BeyoECL Plus kit (P0018S, Beyotime) and quantified using Image J software (NIH, Bethesda, MA, USA), with α‐tubulin protein as the internal reference.

### 2.9 Nuclear Cytoplasmic Separation Assay

Nuclel‐Cyto‐Mem preparation kit (P1201, Applygen, Beijing, China) was used to isolate cytoplasm and nucleus from VSMCs. Subsequently, RNA was extracted from the cytoplasm and nucleus for quantitative real‐time polymerase chain reaction (qRT‐PCR), with GAPDH as the cytoplasmic control transcript and U6 as the nuclear control transcript.

### 2.10 RNA Immunoprecipitation

RNA immunoprecipitation (RIP) was performed using the Magna RIP RNA binding protein immunoprecipitation kit (17‐701, Sigma–Aldrich, St. Louis, MO, USA). Briefly, VSMCs were lysed using RIP lysis buffer, and the cell lysate was incubated with RNA magnetic beads conjugated with KDM1A antibody (1:100, ab129195, Abcam) or negative control IgG antibody (1:100, ab172730, Abcam). The enrichment level of PVT1 was detected by qRT‐PCR, and the primer sequences are shown in Table 1.

### 2.11 Chromatin Immunoprecipitation (ChIP)

Chromatin immunoprecipitation (ChIP) analysis was conducted using the EZ‐Magna ChIP A chromatin immunoprecipitation kit (17‐408, Sigma–Aldrich), with IgG (1:100, ab172730, Abcam) as the negative control. Briefly, VSMCs were cross‐linked in 1% formaldehyde for 10 min and then incubated with 0.125 M glycine at room temperature for 5 min to terminate cross‐linking. Subsequently, the cells were collected for ultrasonic shearing. The lysate diluted with ChIP buffer was immunoprecipitated overnight at 4°C using IgG, anti‐KDM1A (1:20, ab129195, Abcam), and H3K9me2 (1:30, ab176882, Abcam). The antibody‐chromatin complex was precipitated using ChIP‐blocking protein G agarose at 4°C for 2 h, followed by rinsing and elution. After purification, qRT‐PCR was performed. ALOX5 promoter primer sequences are shown in Table 1.

### 2.12 Bioinformatics

The subcellular localization of PVT1 was predicted through the lncATLAS database (https://lncatlas.crg.eu/) [21]. The binding between PVT1 and KDM1A was predicted through the RPISeq database (http://rna.sysu.edu.cn/chipbase/) [22].

### 2.13 Statistical Analysis

Data analysis and map plotting were performed using SPSS 21.0 (IBM Corp., Armonk, NY, USA) and GraphPad Prism 8.0 (GraphPad Software Inc., San Diego, CA, USA). The data were examined for normal distribution and homogeneity of variance. The count data are expressed in cases and percentages, and chi‐square test was used to compare two sets of count data. The measurement data are expressed as mean ± standard deviation. The *t*‐test was adopted for comparisons between two groups, and one‐way or two‐way analysis of variance (ANOVA) was employed for the comparisons among multiple groups, following Tukey's multiple comparison test. The diagnostic value of PVT1 expression for IA was analyzed using the receiver operating curve (ROC). A value of *p* < 0.05 indicated a significant difference.

## 3 Results

### 3.1 Clinical Baseline Characteristics of the Enrolled Population

In the enrolled population of this study, there were no statistically significant differences in age, gender, body mass index (BMI), and history of hypertension between the IA group and the HC group (all *p* > 0.05) (Table 2).

**TABLE 2:** Comparison of clinical baseline characteristics.

Clinical parameters	HC (*N* = 90)	IA (*N* = 90)	*p*
Age	53.53 ± 7.50	52.89 ± 6.60	0.541^a^
Gender (male)	47 (52.2%)	41 (45.6%)	0.371^b^
BMI (kg/m^2^)	23.71 ± 1.98	23.55 ± 1.67	0.558^a^
Hypertension	30 (33.3%)	41 (45.6%)	0.093^b^
Size of aneurysm (mm)	—	10.36 ± 3.61	—
Location of aneurysm			
ICA	—	23 (25.6%)	—
MCA	—	33 (36.7%)	—
ACA/Pcom/posterior	—	34 (37.8%)	—

*Note:* The data are expressed as mean ± standard deviation or *n* (%); *p* < 0.05 is indicative of a statistical difference.

Abbreviations: ACA: anterior cerebral artery; BMI: body mass index; ICA: internal carotid artery; MCAL: middle cerebral artery; Pcom: posterior communicating artery; posterior: posterior circulation (including the vertebral artery, basilar artery, cerebellar arteries, and posterior cerebral artery).

^a^ Independent sample *t*‐test.

^b^ Chi‐square test.

### 3.2 PVT1 Expression Is Upregulated in the Serum of IA Patients and Has High Diagnostic Value

**FIGURE 1:**
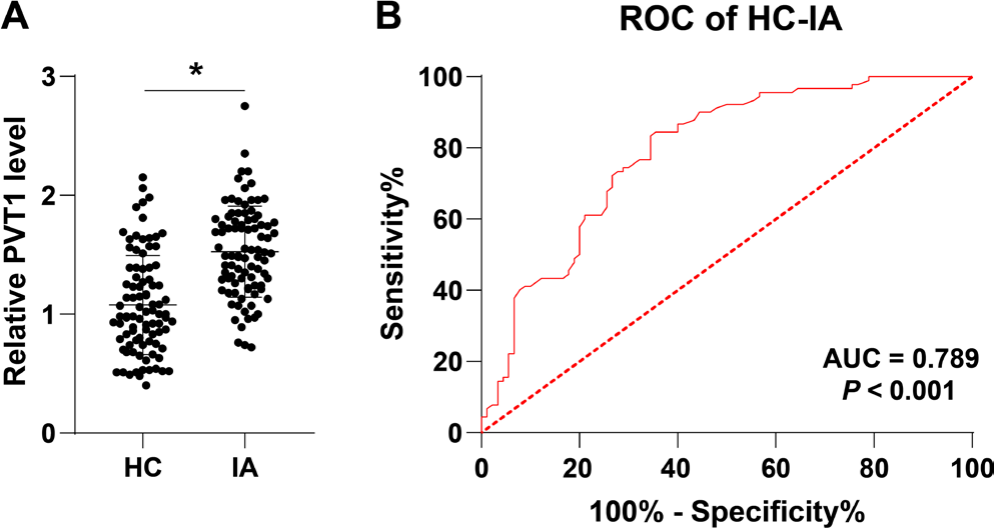
PVT1 expression is upregulated in the serum of IA patients and has high diagnostic value. (A) Detection of PVT1 expression in serum of the healthy control group (*N* = 90) and IA group (*N* = 90) using qRT‐PCR. (B) ROC curve analysis of the diagnostic efficacy of PVT1 in IA patients. Data in panel (A) were analyzed using independent sample *t*‐test, while data in panel (B) were subjected to ROC analysis. *p < 0.05.

We detected the expression of PVT1 in the serum of the HC group and the IA group, and found that the PVT1 expression in the IA group was notably higher than that in the HC group (*p* < 0.05, Figure 1A). Furthermore, we plotted the ROC curve for the diagnosis of IA using PVT1 expression. The results demonstrated that the area under the curve (AUC) for distinguishing the HC and IA groups using PVT1 expression was 0.789, with a cut‐off value of 1.16, a sensitivity of 84.4%, and a specificity of 64.6% (*p* < 0.05, Figure 1B), indicating that the serum PVT1 expression has potential value in assisting the diagnosis of IAs.

### 3.3 Correlation Between PVT1 Expression and Clinical Pathological Characteristics of IA Patients

We assigned IA patients into the PVT1 high‐expression group and PVT1 low‐expression group based on the median value of serum PVT1 expression to further analyze the correlation between serum PVT1 expression and clinical pathological characteristics of IA patients. The results revealed that the serum PVT1 expression was not significantly correlated with age, gender, BMI, and history of hypertension in IA patients (all *p* > 0.05) but was significantly correlated with the size and location of IAs in IA patients (all *p* < 0.05), as shown in Table 3.

**TABLE 3:** Correlation between PVT1 expression and clinical pathological characteristics of IA patients.

Clinical parameters	PVT1 expression		*p*
	Low (*N* = 45)	High (*N* = 45)	
Age	52.31 ± 6.25	53.47 ± 6.95	0.409^a^
Gender (male)	19 (42.2%)	22 (48.9%)	0.525^b^
BMI (kg/m^2^)	23.55 ± 1.37	23.55 ± 1.94	0.988^a^
Hypertension	20 (44.4%)	21 (46.7%)	0.832^b^
Size of aneurysm (mm)	9.26 ± 3.04	11.45 ± 3.83	0.003^a^
Location of aneurysm			0.028^b^
ICA	15 (33.3%)	8 (17.8%)	
MCA	19 (42.2%)	14 (31.1%)	
ACA/Pcom/posterior	11 (24.4%)	23 (51.1%)	

*Note:* The data are expressed as mean ± standard deviation or *n* (%); *p* < 0.05 is indicative of a statistical difference.

^a^ Independent sample *t*‐test.

^b^ Chi‐square test.

### 3.4 Inhibition of PVT1 Restrains Phenotypic Switching of VSMCs

**FIGURE 2:**
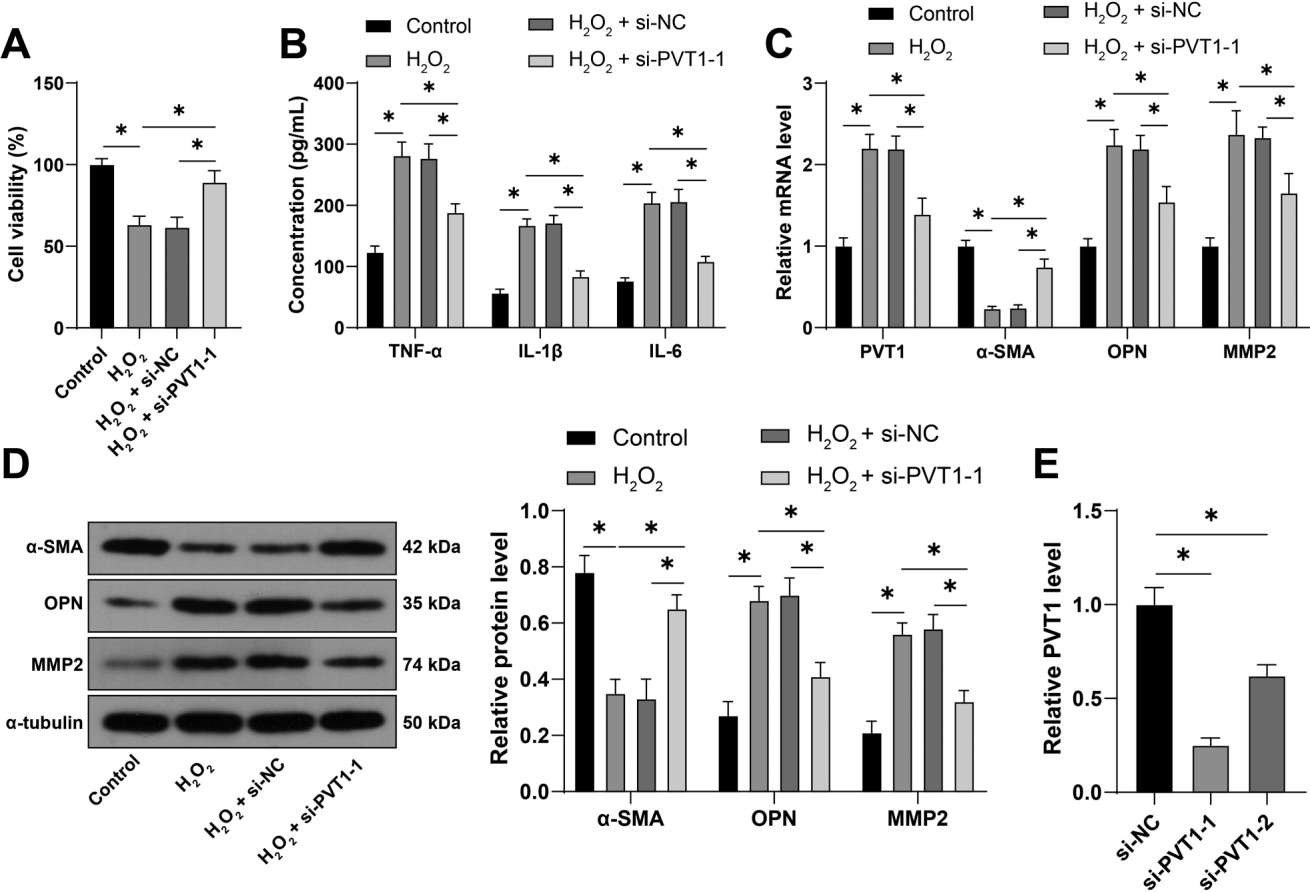
Inhibition of PVT1 inhibits phenotypic switching of VSMCs. siRNA targeting PVT1 (si‐PVT1‐1, si‐PVT1‐2) was transfected into VSMCs, with si‐NC as a control. The transfection efficiency was detected after 48 h, and VSMCs were treated with 0.5 mM H_2_O_2_ for 24 h to induce phenotypic switching. (A) Detection of cell viability using CCK‐8 assay. (B) Detection of inflammatory factors TNF‐α, IL‐1β, and IL‐6 in cells using ELISA. (C) Detection of PVT1, α‐SMA, OPN, and MMP2 expressions using qRT‐PCR. (D) Detection of α‐SMA, OPN, and MMP2 expressions using Western blot. (E) Detection of siRNA transfection efficiency using qRT‐PCR and si‐PVT1‐1 was chosen for subsequent experiments. The cell experiments were repeated 3 times independently. Data were presented as mean ± standard deviation. Data in panels (A)/(E) were analyzed by one‐way ANOVA and data in panels (B)/(C)/(D) were analyzed by two‐way ANOVA, followed by Tukey's multiple comparisons tests. *p < 0.05.

We established a cell model of IA in vitro by treating VSMCs with 0.5 mM H_2_O_2_. H_2_O_2_ treatment resulted in a decrease in cell viability (*p* < 0.05, Figure 2A) and a significant increase in the levels of inflammatory factors TNF‐α, IL‐1β, and IL‐6 (*p* < 0.05, Figure 2B). Compared with the control group, the expression of α‐SMA in the H_2_O_2_ treatment group was significantly declined, while the expressions of OPN and MMP2 were notably increased (*p* < 0.05, Figure 2C,D). Also, PVT1 expression was significantly elevated in H_2_O_2_‐treated cells (*p* < 0.05, Figure 2C). These results indicated that H_2_O_2_ effectively induced VSMCs to transition from a contractile phenotype to a synthetic phenotype and significantly elevated the PVT1 expression. To investigate the role of PVT1 in VSMC phenotypic switching, we inhibited PVT1 expression in VSMCs (*p* < 0.05, Figure 2C,E). Inhibition of PVT1 led to an enhancement in cell viability (*p* < 0.05, Figure 2A), a significant reduction in the levels of inflammatory factors TNF‐α, IL‐1β, and IL‐6 (*p* < 0.05, Figure 2B), a significant increase in α‐SMA expression, and a significant decrease in OPN and MMP2 expressions (*p* < 0.05, Figure 2C,D). These results indicate that inhibition of PVT1 effectively suppresses the phenotypic switching of VSMCs induced by H_2_O_2_.

### 3.5 PVT1 Recruits KDM1A to Reduce H3K9me2 Modification and Thereby Promote ALOX5 Expression

**FIGURE 3:**
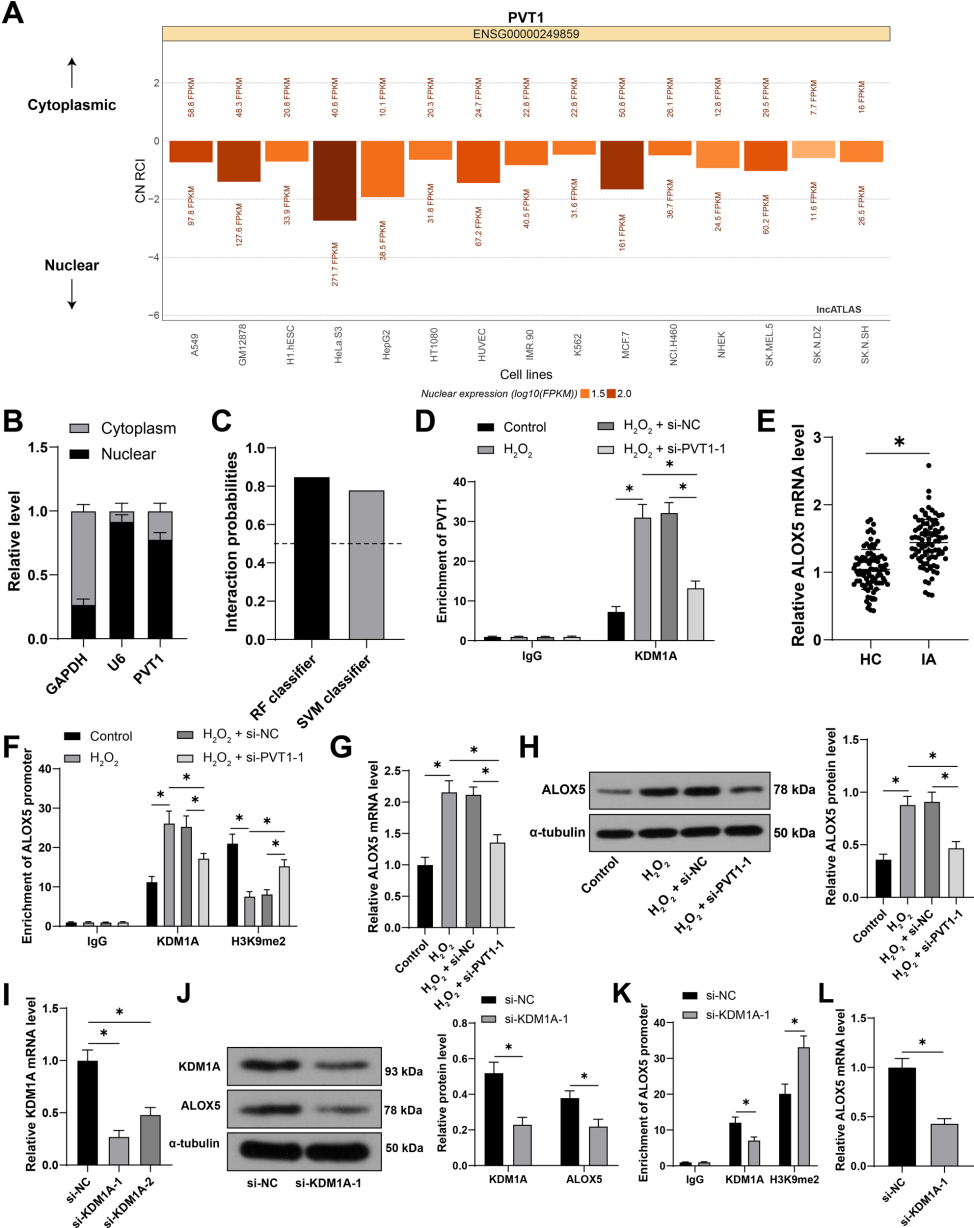
PVT1 recruits KDM1A to reduce H3K9me2 modification and thereby promote ALOX5 expression. (A) Prediction of the subcellular localization of PVT1 through the lncATLAS database. (B) Detection of the subcellular localization of PVT1 through nuclear cytoplasmic separation assay. (C) Prediction of the binding between PVT1 and KDM1A through the RPISeq database. (D) Detection of the binding between PVT1 and KDM1A through RIP assay. (E) Detection of ALOX5 expression in serum of healthy control group (*N* = 90) and IA group (*N* = 90) using qRT‐PCR. (F) Detection of KDM1A and H3K9me2 enrichment on ALOX5 promoter using ChIP. (G, H) Detection of ALOX5 expression using qRT‐PCR and Western bot. siRNA targeting KDM1A (si‐KDM1A‐1, si‐KDM1A‐2) was transfected into VSMCs, with si‐NC as a control. (I) Detection of siRNA transfection efficiency using qRT‐PCR and si‐KDM1A‐1 was chosen for subsequent experiments. (J) Detection of KDM1A and ALOX5 expressions using Western blot. (K) Detection of the enrichment of KDM1A and H3K9me2 on the ALOX5 promoter using ChIP. (L) Detection of ALOX5 expression using qRT‐PCR. The cell experiments were repeated 3 times independently. Data were presented as mean ± standard deviation. Data in panels (E)/(L) were analyzed by *t*‐test. Data in panels (D)/(F)/(J)/(K) were analyzed by two‐way ANOVA, and data in panels (G)/(H)/(I) were analyzed by one‐way ANOVA, followed by Tukey's multiple comparisons test. *p < 0.05.

We predicted through the lncATLAS database that PVT1 is mainly distributed in the nucleus (Figure 3A) and validated the subcellular localization of PVT1 through nuclear cytoplasmic separation assay (Figure 3B), indicating that PVT1 may play a transcriptional regulatory role in the nucleus. KDM1A, as an important histone demethylase, promotes downstream gene expression by removing H3K9me2 modification [23]. We predicted the binding potential between PVT1 and KDM1A through the RPISeq database (Figure 3C) and validated the binding between PVT1 and KDM1A through RIP assay. In H_2_O_2_‐induced VSMCs, the binding between PVT1 and KDM1A was enhanced, but weakened after inhibition of PVT1 (*p* < 0.05, Figure 3D). ALOX5 expression was elevated in IA patients [24], and we obtained the same results in clinical samples (*p* < 0.05, Figure 3E). Therefore, we speculated that the binding of PVT1 to KDM1A reduced H3K9me2 modification to elevate ALOX5 expression. ChIP assay revealed that both KDM1A and H3K9me2 were significantly enriched on the ALOX5 promoter. H_2_O_2_‐induced VSMCs showed an increase in KDM1A enrichment, while inhibition of PVT1 led to a decrease in KDM1A enrichment and an increase in H3K9me2 (*p* < 0.05, Figure 3F). In addition, ALOX5 expression was upregulated in H_2_O_2_‐induced VSMCs and significantly diminished after inhibition of PVT1 (*p* < 0.05, Figure 3G,H). We further inhibited KDM1A expression in VSMCs (*p* < 0.05, Figure 3I,J), which resulted in a decrease in the enrichment of KDM1A on the ALOX5 promoter, an increase in the enrichment of H3K9me2, and a significant inhibition of ALOX5 expression (*p* < 0.05, Figure 3K,L,J). The above results indicate that PVT1 promotes ALOX5 expression by recruiting KDM1A to reduce H3K9me2 modification.

### 3.6 Overexpression of ALOX5 Abolishes the Inhibitory Effect of PVT1 Silencing on VSMC Phenotypic Switching

**FIGURE 4:**
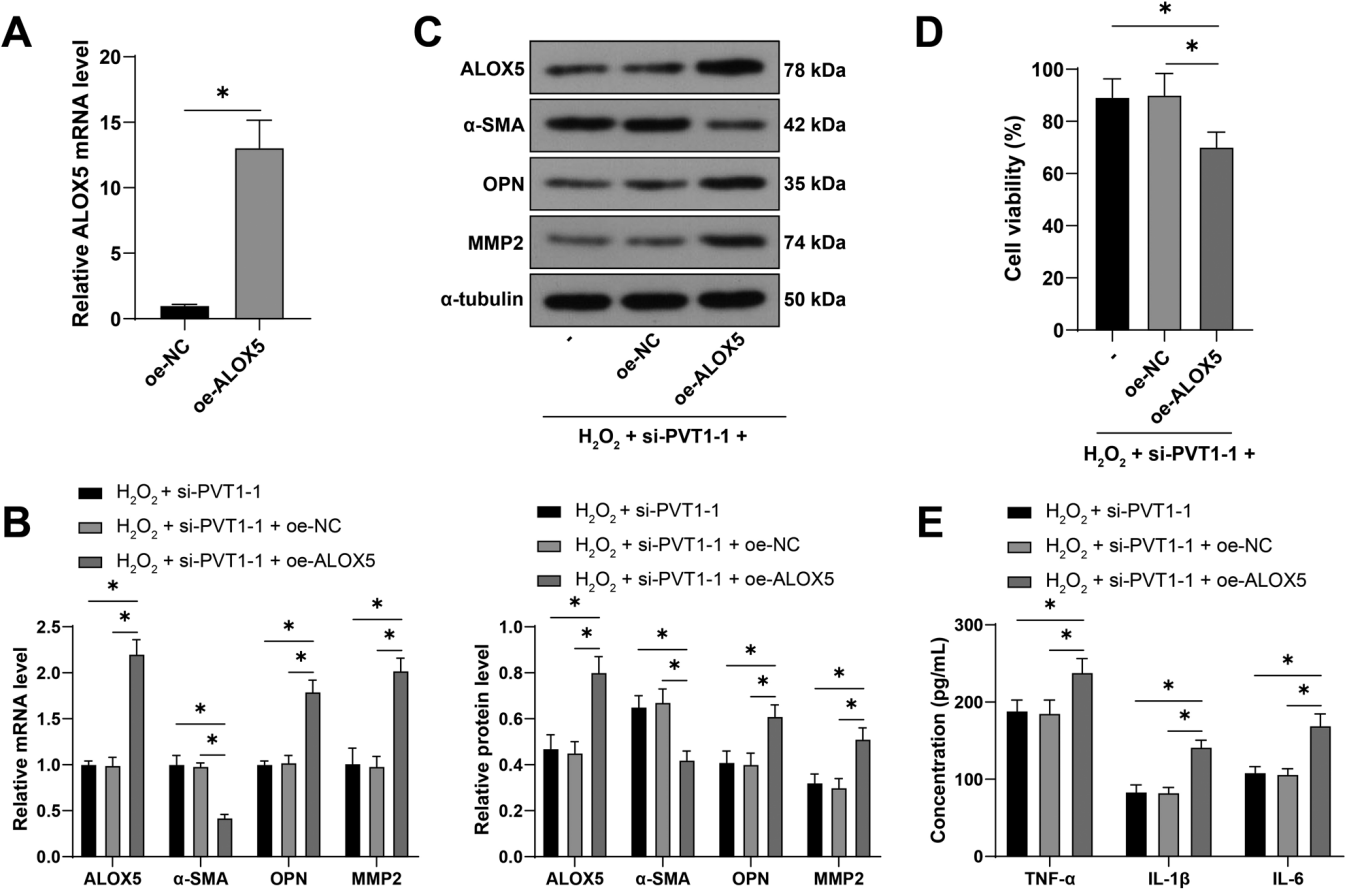
Overexpression of ALOX5 abolishes the inhibitory effect of PVT1 silencing on VSMC phenotypic switching. ALOX5 overexpression plasmid (oe‐ALOX5) was transfected into VSMCs, with oe‐NC as a control. The transfection efficiency was detected after 48 h, and VSMCs were treated with 0.5 mM H_2_O_2_ for 24 h to induce phenotypic switching. (A) Detection of transfection efficiency using qRT‐PCR. (B, C) Detection of ALOX5, α‐SMA, OPN, and MMP2 expressions using qRT‐PCR and Western blot. (D) Detection of cell viability using CCK‐8 assay. (E) Detection of inflammatory factors TNF‐α, IL‐1β, and IL‐6 in cells using ELISA. The cell experiments were repeated 3 times independently. Data were presented as mean ± standard deviation. Data in panel (A) were analyzed by *t*‐test. Data in panels (B)/(C)/(E) were analyzed by two‐way ANOVA, and data in panel (D) were analyzed by one‐way ANOVA, followed by Tukey's multiple comparisons tests. *p < 0.05.

To verify the above mechanism, we overexpressed ALOX5 in VSMCs and conducted a combined experiment with si‐PVT1‐1 (*p* < 0.05, Figure 4A–C). Compared with the inhibition of PVT1 alone, the combined treatment resulted in a significant reduction in cell viability (*p* < 0.05, Figure 4D), a significant increase in inflammatory factors TNF‐α, IL‐1β, and IL‐6 (*p* < 0.05, Figure 4E), and a significant decrease in α‐SMA expression, as well as a significant increase in OPN and MMP2 expressions (*p* < 0.05, Figure 4B,C), promoting the transition of VSMCs from a contractile to a synthetic phenotype. These results indicate that the overexpression of ALOX5 partially abolishes the inhibitory effect of PVT1 silencing on VSMC phenotypic switching.

## 4 Discussion

**FIGURE 5:**
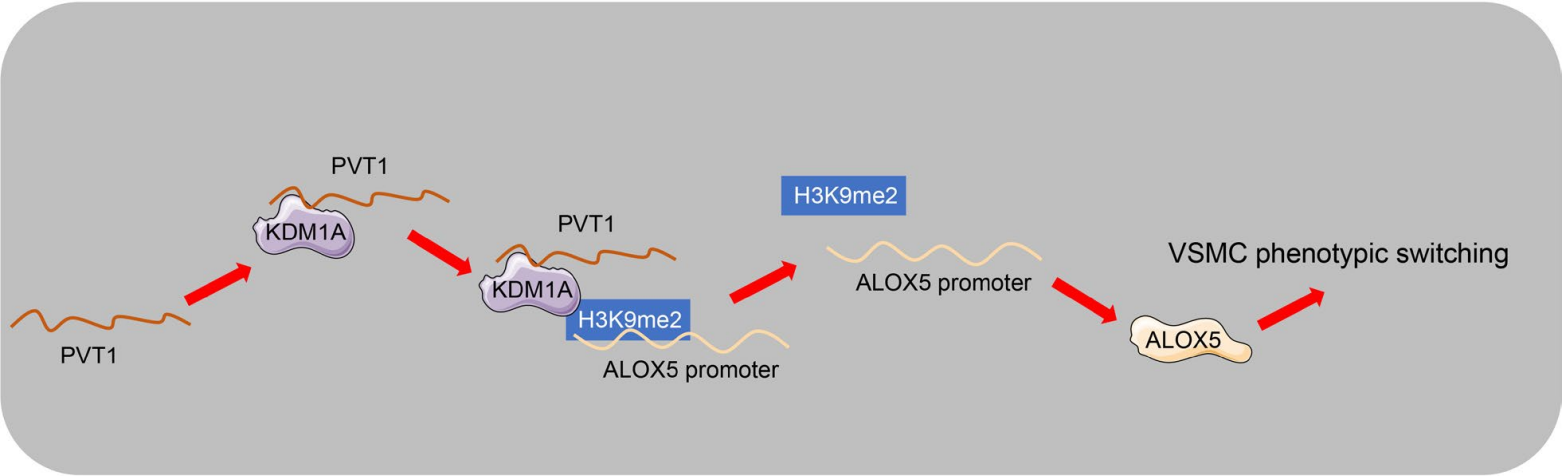
PVT1 promotes VSMC phenotypic switching by recruiting KDM1A to reduce H3K9me2 modification and enhance ALOX5 expression.

IA lingers as a catastrophic clinical event. The combination of ECM destruction, endothelial dysfunction, VSMC phenotypic switching, and inflammation represents a dynamic process leading to vascular wall deterioration and subsequent IA formation [25, 26]. Compelling evidence has established the critical role of lncRNAs in the diagnosis and treatment of IAs, as well as the prediction of high risk of rupture [9]. This study demonstrates that lncRNA PVT1 is highly expressed in IAs and promotes the phenotypic switching of VSMCs (Figure 5).

LncRNA PVT1 plays a vital role in IA formation by forming a competitive endogenous RNA network and manipulating the PI3K‐Akt signaling pathway [10]. Bioinformatics analysis has also identified PVT1 as a ferroptosis‐related lncRNA implicated in IA formation [11]. PVT1 augments inflammation and pyroptosis of VSMCs in abdominal aortic aneurysm by repressing miR‐186‐5p expression [27]. PVT1 can stimulate the expressions of COL1A1 and COL5A2, two genes associated with collagen formation that lead to ECM remodeling of intracranial blood vessels, thereby promoting IA formation [28]. We detected PVT1 expression in the serum of HC and IA groups and found that PVT1 was notably upregulated in IA patients. Serum PVT1 expression possessed certain potential value in assisting the diagnosis of IAs. In addition, PVT1 expression was evidently correlated with the size and location of IAs. The roles of other lncRNAs in IA formation and their interaction with PVT1 are also worth exploring.

VSMCs exhibit significant plasticity and undergo profound phenotypic switching during vascular injury repair and in response to changes in blood flow [6]. The transformation of VSMCs into a synthetic phenotype is a prerequisite for the onset of various cardiovascular diseases, including IAs [4]. This process involves a decrease in contractile proteins such as α‐SMA, as well as an increase in inflammatory factors such as matrix metalloproteinases and TNF‐α. We treated VSMCs with 0.5 mM H_2_O_2_ to establish an IA cell model in vitro and found that H_2_O_2_ treatment weakened the viability of VSMCs, elevated the levels of inflammatory factors (TNF‐α, IL‐1β, IL‐6), reduced the α‐SMA expression, and increased the OPN and MMP2 expressions, accompanied by a significant upregulation of PVT1 expression. These results indicate that H_2_O_2_ effectively induced the transition of VSMCs from contractile phenotype to synthetic phenotype and significantly elevated PVT1 expression. LncRNA PVT1 facilitates VSMCs stimulated by angiotensin II to switch from a contractile phenotype to a synthetic phenotype [12]. LncRNA PVT1 modulates the phenotypic switching and migration of VSMCs by mediating glycolysis in decidual spiral artery remodeling [29]. Overexpression of PVT1 is also associated with the synthetic phenotype of stent‐stimulated VSMCs [30]. Consistently, we found that inhibition of PVT1 effectively suppressed the phenotypic switching of VSMCs induced by H_2_O_2_. These findings suggest the necessity of the development of PVT1‐based biomarker detection methods or small‐molecule inhibitors targeting PVT1 or its downstream pathways for the management of IAs.

Thereafter, we attempted to determine the exact mechanism of PVT1 affecting VSMC phenotypic switching. We predicted the binding potential between PVT1 and KDM1A through the RPISeq database and verified the binding relationship between PVT1 and KDM1A via RIP assay. KDM1A can specially demethylate lysine residues of histone H3K4me1/2 and H3K9me1/2. Silencing of KDM1A prevents VSMCs from transforming into a synthetic phenotype and represses the progression of IAs [16, 17]. In arteries undergoing injury‐induced remodeling, the H3K9me2 level in VSMCs is declined while inflammation is intensified [18]. ALOXs are the main regulatory factors catalyzing the conversion of polyunsaturated fatty acids into lipid hydroperoxides, leading to cell membrane damage and ferroptosis [31]. Elevated ALOX5 has been demonstrated to increase the risk of atherosclerosis and myocardial infarction [32, 33, 34]. Importantly, ALOX5 expression in aneurysm wall tissues is obviously increased [35]. ALOX5 is the most significantly differentially expressed gene related to oxidative stress in the pathogenesis of IAs [24]. ALOX5 expression was elevated in the clinical IA samples we collected, upregulated in H_2_O_2_‐induced VSMCs, and significantly declined after inhibition of PVT1. Therefore, we speculated that the binding of PVT1 to KDM1A in H_2_O_2_‐induced VSMCs reduced H3K9me2 modification and promoted ALOX5 expression. ChIP assay confirmed that both KDM1A and H3K9me2 were significantly enriched on the ALOX5 promoter. H_2_O_2_‐induced VSMCs showed an increase in KDM1A enrichment, while inhibition of PVT1 led to a decrease in KDM1A enrichment and an increase in H3K9me2. Further inhibition of KDM1A expression in VSMCs resulted in reduced enrichment of KDM1A on the ALOX5 promoter, increased enrichment of H3K9me2, and significant inhibition of ALOX5 expression. The above results indicated that PVT1 promoted ALOX5 expression by recruiting KDM1A to reduce H3K9me2 modification. It is indicated that utilizing siRNA or antisense oligonucleotides to inhibit PVT1 expression or developing small‐molecule inhibitors targeting KDM1A may be potential therapeutic strategies for IAs.

This study has certain limitations. Firstly, the number of cases included in this study is relatively small, and further expansion of the sample size and multi‐center research are needed. Secondly, this study is limited to the cellular level and does not involve pathological tissues or animal experiments. Thirdly, our group conditions do not support us in conducting gene microarray analysis on IA patients, so we cannot conduct more in‐depth bioinformatics analysis on differentially expressed gene data currently. Also, we failed to perform RNA‐seq or proteomics to identify broader networks regulated by PVT1. Previous reports [10, 11] have conducted GO and KEGG analysis on differentially expressed genes in IAs, and the results show that differentially expressed genes are mainly enriched in collagen and extracellular mechanism‐related biological processes and the PI3K‐AKT signaling pathway. Moreover, the mechanism by which PVT1 regulates VSMC phenotypic switching is relatively simple. It is necessary to explore other potential regulatory mechanisms of PVT1. In the future, we will conduct multi‐center prospective studies, expand the sample size, and match controls to increase the credibility of the results. In addition, we will further validate the mechanism of PVT1‐KDM1A‐ALOX5 in animals and explore other regulatory mechanisms of PVT1 in VSMC phenotypic switching. More in‐depth research is needed to promote the clinical translation using RNA interference or small‐molecule inhibitors targeting PVT1.

## 5 Conclusion

PVT1 expression is correlated with the size and location of IAs, and PVT1 elevates ALOX5 expression by recruiting KDM1A to remove H3K9me2 modification, thereby facilitating VSMC phenotypic switching. It is indicated that utilizing siRNA or antisense oligonucleotides to inhibit PVT1 expression or developing small‐molecule inhibitors targeting KDM1A may be potential therapeutic strategies for IAs.

## Conflicts of Interest

The authors declare no conflicts of interest.

## Data Availability

The data that support the findings of this study are available from the corresponding author upon reasonable request.
